# Editorial

**DOI:** 10.31372/20200504.1123

**Published:** 2021

## Aloha and Mahalo

Defined by Andrews and Parker (1922, p. 52), “aloha” in Hawaiian is “A word expressing different feelings; love, affection, gratitude, kindness, pity, compassion, grief, the modern common salutation at meeting; parting.” It has another deeper spiritual significance related to love compassion, sympathy, mercy, and peace. Aloha says goodbye to the past year of chaos and crisis and welcome 2021 to peace, compassion, and new beginnings.

Despite personnel problems and production issues due to the pandemic, we will complete publication of all 2020 issues by March of this year. As we face a change in our distributor due to the economic downturn, we look forward with expectations for a new editor and distributor. Our recent member survey highlighted membership support in favor of the organization having a journal and individuals were willing to support it with their membership fees and some willing to add to that support with their own finances. Because of this mandate we will continue to explore ways to produce the journal. A seasoned editor will be leading the new task force to determine where to go from here.

Looking back on the bright side, we offered our first student scholarship for best abstract. Congratulations to the honoree! Other good news is the re-indexing by SCOPUS in addition to EBSCO, PMC, and DOAJ. Finally, we have had consistent submissions by members and nonmembers and for a small society and fledging journal we are doing all right.

As founding editor and editor-in-chief for the past six years, I’m proud of how the journal has progressed despite our many challenges and happy that it provides a venue for our members and nonmembers to contribute to knowledge regarding research, practice, education, and policy on Asian and Pacific Islanders.

Pukui and Elbert (1986) translate “mahalo” to thanks, gratitude, admiration, praise, esteem, regards, or respects. It has been an honor serving as founding editor-in-chief and I want to extend a sincere mahalo to UH Press, especially Pam Wilson; our editorial board; our associate editor, Patricia Alpert; our special issues guest editors, Eun-Ok Im, Merle Kataoka-Yahiro, May Kealoha, Mijung Park, Reimund Serifica, Seonae Yeo; and the entire AAPINA membership for supporting the journal these past years.

Wishing everyone a safe, healthy, and happy 2021. Aloha nui loa and A hui hou kakou.

Jillian Inouye, PhD, FAAN

Editor-in-Chief
